# Supplementary material for: Improving the Reliability of Muscle Tissue Characterization Post-Stroke: A Secondary Statistical Analysis of Echotexture Features
Source: J Clin Med. 2025 Apr 23;14(9):2902. doi: 10.3390/jcm14092902 (PMC12072403; doi:10.3390/jcm14092902)

SUPPLEMENTARY MATERIAL

Table S1. Features derived from first-order statistics.

Feature	Fermula
Echogenicity (Echointensity)	$mean(image)$
Variance (var)	$\frac{1}{N}\sum_{i=1}^{N}(x_i - \mu)^2$
Standard Deviation (std)	$\sqrt{var(image)}$
Echovariation	$\frac{std(image) * 100}{mean(image)}$
Skewness	$\frac{\sum_{i=1}^{N}(x_i - \mu)^3}{(N-1)\sigma^3}$
Kurtosis	$\frac{\sum_{i=1}^{N}(x_i - \mu)^4}{(N-1)\sigma^4}$

Table S2. Features derived from gray-level co-occurrence matrix.

Feature	Fermula
Correlation [10]	$\frac{\sum_{i,j}(i-\mu_i)(j-\mu_j)P(i,j)}{\sigma_i\sigma_j}$
Dissimilarity [10]	$\sum_{i,j}\lvert i-j\rvert P(i,j)$
Contrast [10]	$\sum_{i,j}(i-j)^2P(i,j)$
Homogeneity [10]	$\sum_{i,j}\frac{P(i,j)}{1+\lvert i-j\rvert}$
Angular Second Moment (ASM) [10]	$\sum_{i,j}P(i,j)^2$
Energy [10]	$\sqrt{\sum_{i,j}P(i,j)^2}$
Max Probability [10]	$\max\,(P(i,j))$
Entropy [10]	$-\sum_{i,j}P(i,j)\log\,(P(i,j))$
Cluster Shade [33]	$\sum_{i,j}\left(i+j-\mu_x-\mu_y\right)^3P(i,j)$
Cluster Prominent [33]	$\sum_{i,j}\left(i+j-\mu_x-\mu_y\right)^4P(i,j)$

Table S3. Features derived from the gray-level run-length matrix.

Feature	Fermula
Short Run Emphasis (SRE) [12]	$\frac{1}{N}\sum_{i,j}\frac{P(i,j)}{j^2}$
Long Run Emphasis (LRU) [12]	$\frac{1}{N}\sum_{i,j}P(i,j)j^2$
Gray Level Uniformity (GLU) [34]	$\sum_{i,j}P(i,j)^2$
Run Length Uniformity (RLU) [34]	$\sum_{j}(\sum_{i}P(i,j))^2$
Run Percentage (RPC) [35]	$\frac{Number\ of\ Runs}{Number\ of\ Pixel}$

Table S4. Impairment-level agreement among examiners.

	Cohen´s Kappa (95%CI)
**Main outcomes**	
Echointensity	1 (1, 1)
Echovariation	1 (1, 1)
**Secondary outcomes**	
Dissimilarity	1 (1, 1)
Entropy	1 (1, 1)
GLU	1 (1, 1)
Homogeneity	1 (1, 1)
Kurtosis	1 (1, 1)
RLU	1 (1, 1)
RPC	1 (1, 1)
Contrast	0.90 (0.70, 1)
Correlation	0.90 (0.70, 1)
Maximum Probability	0.89 (0.69, 1)
SRE	0.89 (0.69, 1)
Standard deviation	0.83 (0.50, 1)
Cluster shade	0.80 (0.54, 1)
LRE	0.80 (0.54, 1)
Energy	0.77 (0.46, 1)
Cluster prominence	0.71 (0.41, 0.99)
ASM	0.70 (0.40, 0.99)
Skew	0.69 (0.31, 1)
Variance	0.50 (0.05, 0.95)

95%CI: 95% confidence interval.

Table S5. Impairment level vs. modified Heckmatt scale correlation.

Examiner 1		Examiner 2	
	**ρ (95%CI)**		**ρ (95%CI)**
**Main outcomes**			
Echointensity	1 (1, 1)	Echointensity	1 (1, 1)
Echovariation	1 (1, 1)	Echovariation	1 (1, 1)
**Secondary outcomes**			
Cluster prominence	1 (1, 1)	Cluster shade	1 (1, 1)
Cluster shade	1 (1, 1)	Energy	1 (1, 1)
GLU	1 (1, 1)	GLU	1 (1, 1)
Kurtosis	1 (1, 1)	Kurtosis	1 (1, 1)
RPC	1 (1, 1)	LRE	1 (1, 1)
Standard deviation	1 (1, 1)	RPC	1 (1, 1)
Variance	1 (1, 1)	Skew	1 (1, 1)
Skew	1 (1, 1)	SRE	1 (1, 1)
Energy	1 (1, 1)	Standard deviation	1 (1, 1)
SRE	1 (1, 1)	Variance	1 (1, 1)
LRE	1 (1, 1)	Dissimilarity	1 (1, 1)
Homogeneity	1 (1, 1)	Entropy	1 (1, 1)
Maximum Probability	1 (1, 1)	Cluster prominence	1 (1, 1)
RLU	1 (1, 1)	RLU	1 (1, 1)
ASM	0.99 (0.99, 1)	Maximum Probability	1 (1, 1)
Entropy	0.95 (0.80, 0.99)	Homogeneity	1 (1, 1)
Dissimilarity	0.91 (0.66, 0.98)	ASM	0.99 (0.99, 1)
Correlation	0.91 (0.65, 0.98)	Contrast	0.96 (0.82, 0.99)
Contrast	0.90 (0.63, 0.98)	Correlation	0.86 (0.49, 0.97)

95%CI: 95% confidence interval.

Figure S1. Plot diagnosis for impairment-level models for echovariation model.

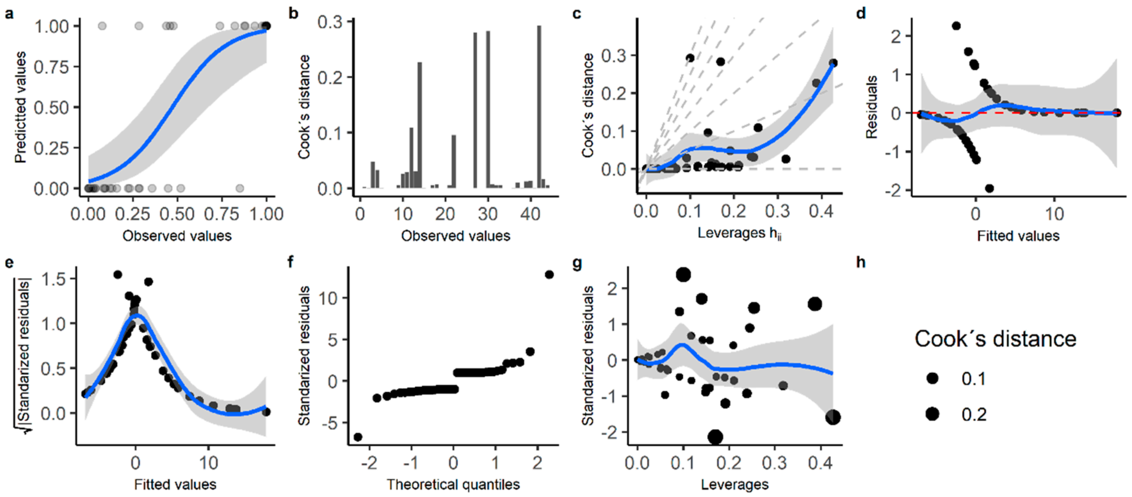

Figure S2. Plot diagnosis for impairment-level models for echointensity model.

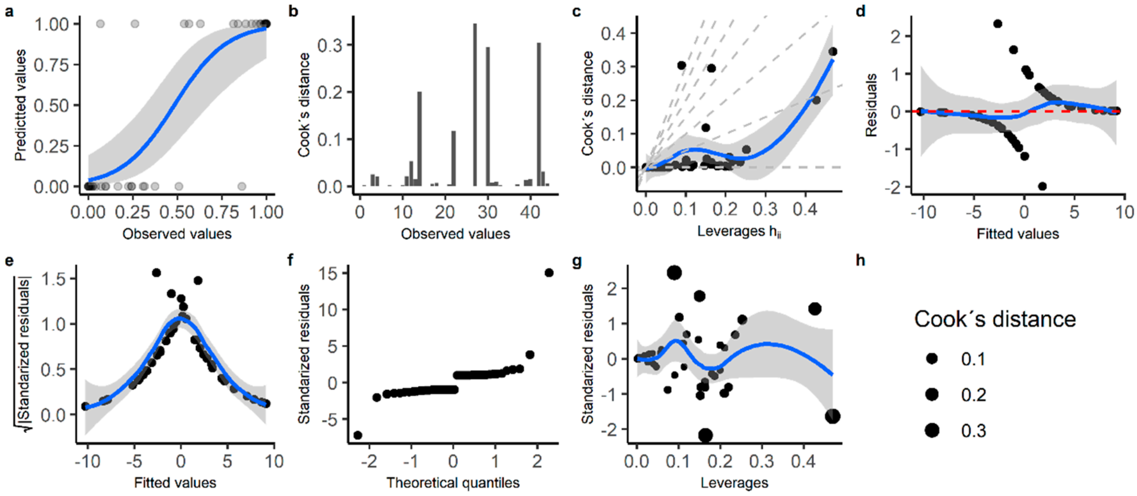

Supplement: Supplementary file 1 [file jcm-14-02902-s001.zip › jcm-3567095-supplementary.pdf]
